# Human airway organoids as a versatile model to study BSL-4 virus replication and pathogenesis

**DOI:** 10.1038/s41598-026-45813-6

**Published:** 2026-03-27

**Authors:** Joo-Hee Wälzlein, Sebastian Reusch, Jenny Ospina-Garcia, Ruth Olmer, Marc A. Schneider, Laura V. Klotz, Christian Klotz, Susann Kummer

**Affiliations:** 1 Centre for Biological Threats and Special Pathogens (BSL-4 Laboratory), Robert Koch-Institute, Berlin, Germany; 2 Unit “Mycotic and Parasitic Agents and Mycobacteria”, Robert Koch-Institute, Berlin, Germany; 3 Department of Cardiothoracic, Transplantation and Vascular Surgery (HTTG), Biomedical Research in Endstage and Obstructive Lung Disease Hannover (BREATH), German Center for Lung Research (DZL), Leibniz Research Laboratories for Biotechnology and Artificial Organs (LEBAO), Hannover Medical School, Hannover, Germany; 4 Translational Research Unit, Thoraxklinik at Heidelberg University Hospital, Heidelberg, Germany; 5 Translational Lung Research Center Heidelberg (TRLC), Member of The German Center for Lung Research (DZL), Heidelberg, Germany; 6 Department of Surgery, Thoraxklinik at Heidelberg University Hospital, Heidelberg, Germany

**Keywords:** BSL-4 viruses, Airway organoids, Human infection model, Biological techniques, Biotechnology, Diseases, Immunology, Microbiology

## Abstract

Research with BSL-4 viruses such as Ebola, Marburg, and Nipah presents significant challenges due to their high virulence and the stringent containment measures required. This study establishes human airway organoids as a robust model for investigating BSL-4 pathogens. In contrast to conventional cell lines, airway organoids enable investigation of virus-host interactions within a human tissue context, providing insights that are more directly translatable to human disease. We generated airway organoids from both clinical donor tissues and commercially available nasal epithelial cells and showed in comparative analyses with whole lung tissue that these organoids are comparable in terms of cell composition. Despite biological variations, airway organoids derived from different sources and donors exhibit a remarkably similar cellular make-up. We further demonstrated that organoids derived from nasal swabs can effectively replicate BSL-4 viruses. This establishes them as a standardized 3D model for broader research applications including infection kinetics, immune evasion, and tissue-specific tropism within a controlled environment. This platform provides a powerful tool for antiviral testing and studying virus-host interactions, thus helping bridge critical gaps in high containment virus research and advancing our understanding of these pathogens, bypassing some of the challenges of animal models.

**Supplementary Information:**

The online version contains supplementary material available at 10.1038/s41598-026-45813-6.

## Introduction

A significant challenge in studying BSL-4 viruses, such as Ebola, Marburg, and Nipah, lies in the limitations and accessibility of animal models. While non-human primates, humanized mice, and species-specific models such as the Syrian hamster for Nipah virus have provided crucial insights into pathogenesis, they are ethically questionable, costly and demand considerable resources when studied under high containment conditions. Moreover, factors such as containment requirements, high costs, and limited access to specialized facilities restrict the number of studies on BSL-4 viruses, thereby limiting our understanding of their pathogenesis and hindering the development of effective therapeutic interventions. Ebola virus causes severe hemorrhagic fever with high mortality rates, primarily in sub-Saharan Africa. It spreads through direct contact with body fluids and can lead to widespread outbreaks, as seen during the 2014–2016 epidemic in West Africa^1^. Marburg virus, a close relative of Ebola, also causes hemorrhagic fever with similar transmission and clinical outcomes. Both viruses belong to the Filoviridae family and are known for their rapid disease progression and high case fatality rates^2^. In contrast, Nipah virus, a member of the Paramyxoviridae family, causes encephalitis and respiratory illness. First identified in Malaysia in 1998, Nipah virus has since caused multiple outbreaks across South and Southeast Asia^3–5^. These zoonotic viruses are typically transmitted to humans from animals such as fruit bats or pigs^5–7^. Currently, there are no specific treatments or vaccines available (with exception of the Ebola vaccine, which is only effective against the Ebola Zaire strain)^8^.

Given the challenges associated with available animal models, human stem cell-derived organoids offer a promising alternative for studying these viruses in a controlled environment that closely resembles human tissue^9^. This approach not only allows for the examination of virus-host interactions but also facilitates the testing of potential therapeutic interventions, providing insights that are more predictive of human outcomes. To provide proof-of-concept, we compared several epithelial organoids derived from respiratory tissue to evaluate cell type conformity using standardized culture protocols and demonstrate the utility of these cultures for studying BSL-4 viruses in a Biosafety Level-4 environment.

Organoids are three-dimensional, self-organizing structures derived from stem cells that better replicate organ physiology compared to 2D or conventional 3D cell cultures using immortalized cell lines^10,11^. While pluripotent stem cell-derived organoids mimic organogenesis, providing high-fidelity models of pathophysiological complexity in organ development, adult stem cell-derived organoids retain organ identity and genetic stability over time^2,12^. Culture protocols for adult stem cell-derived organoids are relatively straightforward and can easily be established in conventional cell culture laboratories. Transcriptomic studies indicate that adult stem cell-derived organoids, including airway organoids, can reproduce key gene expression features of their tissue of origin, which closely align with primary lung tissue profiles^13,14^. However, some studies show that organoid transcriptomes may differ substantially from those of native tissues^15^. Despite these discrepancies, organoids remain valuable models for studying and modelling organ-specific pathologies, although findings should be interpreted with caution given that they are not fully equivalent to native tissues.

## Results

**Fig. 1:**
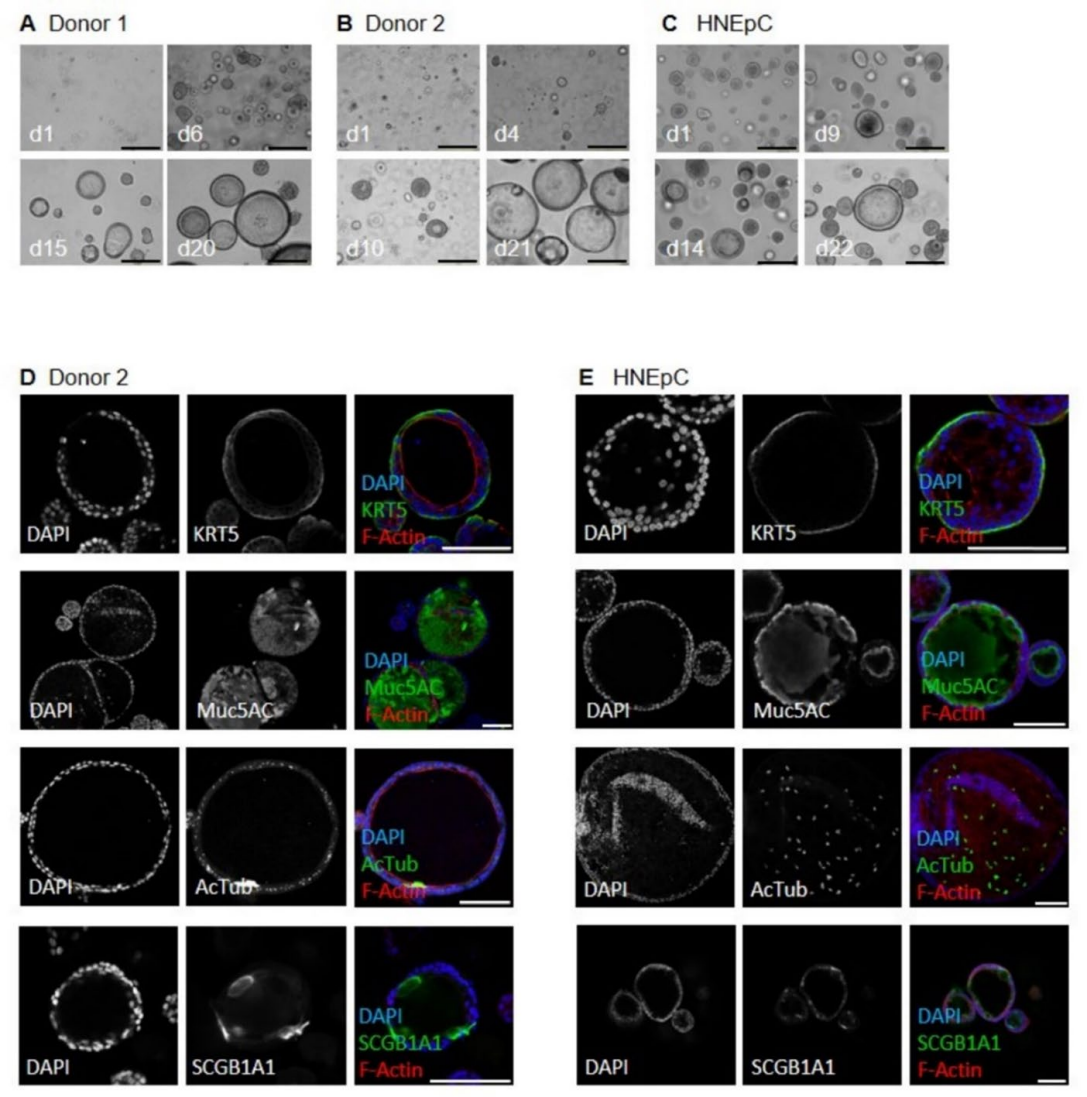
Microscopic analysis of human airway organoids (AOs) focusing on their morphology and the spatial localisation of cellular marker proteins. **A-C**: Bright-field images of AOs at different days of cultivation as indicated. Scale bars = 50 μm. Images were taken employing a widefield microscope and a 20x air objective (d = day). **D/E**: Immunofluorescence staining of AOs derived from adult stem cells of human lung tissue from donor 2 or HNEpC using antibodies specific to keratin5 (KRT5), mucin5AC (Muc5AC), acetylated tubulin (AcTub), and uteroglobin (SCGB1A1). Note that AcTub exhibits off-target staining of spindles of mitotic cells. Cell nuclei were counterstained with DAPI and F-actin with phalloidin, respectively. Image acquisition was performed using a confocal laser scanning microscope and a 20x immersion oil objective. Scale bars = 100 μm.

**Fig. 2:**
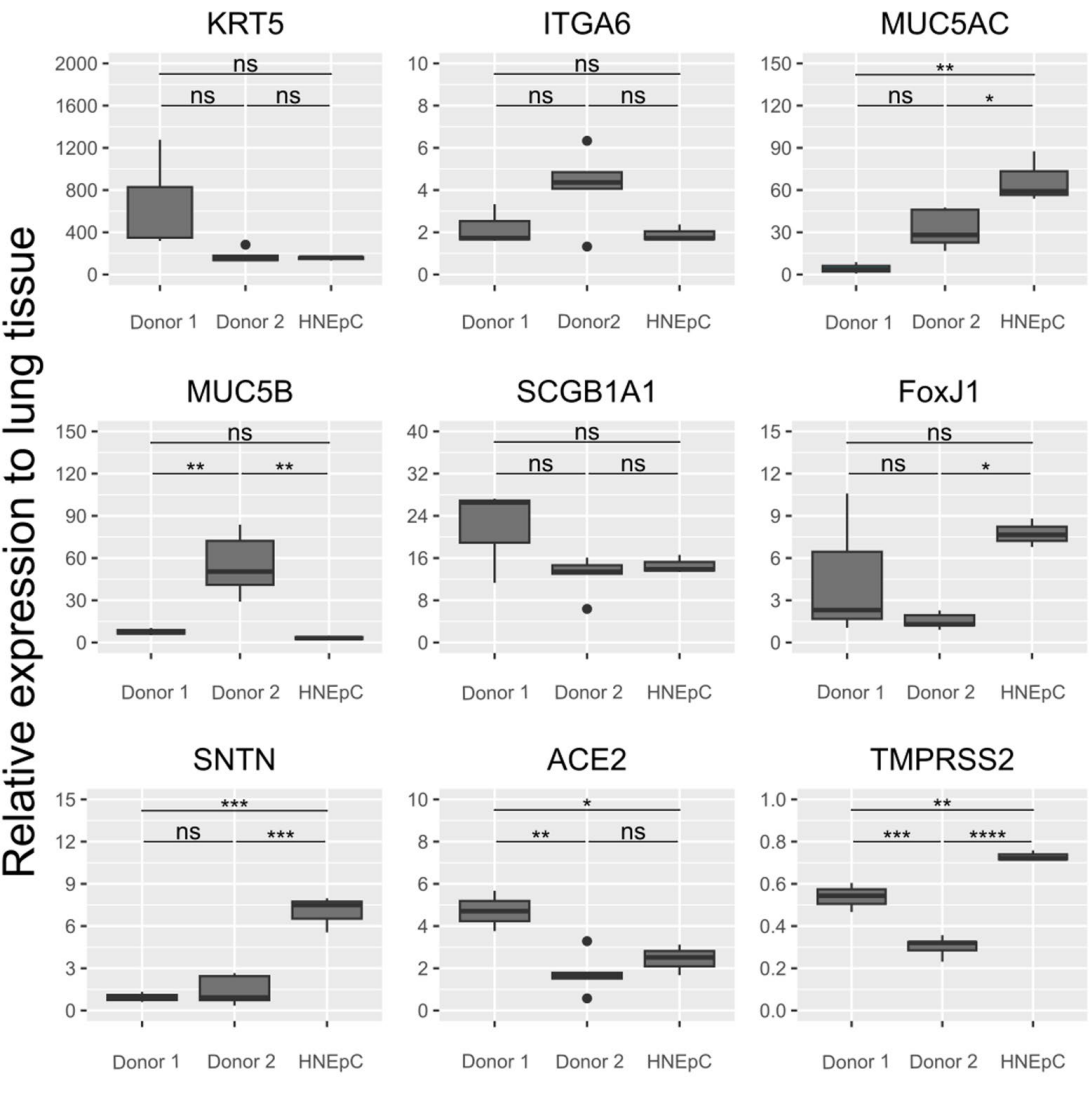
qPCR-analysis of human airway organoids (AOs) reveals expression of all major airway epithelial marker genes comparable to human lung tissue. GAPDH was used as the reference housekeeping gene for normalization. Boxplots show the median and interquartile range of three biological replicates (n = 3), each with two technical replicates. Whiskers represent the highest and lowest values, and outliers are plotted as dots. D1 = donor 1, D2 = donor 2, HNEpC = human nasal epithelial cells. Statistical significance was calculated using one-way ANOVA, followed by a post-hoc Tukey`s test (ns = *p* > 0.05; * = *p* ≤ 0.05; ** = *p* ≤ 0.01; *** = *p* ≤ 0.001; **** = *p* ≤ 0.0001).

We have successfully established airway organoids derived from different sources as reliable infection models for BSL-4 viruses. Ebola, Marburg and Nipah virus were employed in this proof-of-concept study to demonstrate viral replication within the established culture system. Airway organoids from various source material, from donor 1, 2 (both healthy lung tissue) and HNEpCs (commercial nasal epithelial cells) exhibited similar morphology and cellular marker expression typical of human respiratory epithelial tissue (Fig. 1). Cystic and solid structures were observed, with cystic formations, mostly larger than solid structures, characterized by cavities filled with mucus or cell debris becoming larger over time (Fig. 1A-C). Nasal epithelial cell-derived organoids predominantly formed smaller, solid structures, with some cystic formations appearing in long-term cultures (Fig. 1C). Immunofluorescence (IF) and qPCR analysis confirmed the presence and localization of basal cells (keratin 5, KRT5), goblet cells (mucin 5AC, MUC5AC), ciliated cells (acetylated tubulin, AcTUB), and club cells (secretoglobin family 1A member 1, SCGB1A1) in all organoids, consistent with primary airway tissue (Figs. 1D-E and 2)^16^. Specifically, IF of donor 2 and HNEpC-derived organoids revealed comparable staining for all markers. Comprehensive qPCR analysis verified the expression of all major airway epithelial cell type markers, with levels generally comparable to or exceeding those observed in lung tissue (Fig. 2). The presence of basal cells is marked by the expression of KRT5 and integrin alpha-6 (ITGA6), while goblet cells are identified by the expression of MUC5B, MUC5AC, and sentan, cilia apical structure protein (SNTN). Club cells are indicated by SCGB1A1 expression, and ciliated cells are characterized by the expression of forkhead box J1 (FOXJ1) as well as SNTN. Additionally, angiotensin-converting enzyme 2 (ACE2) and transmembrane protease, serine 2 (TMPRSS2) serve as markers for viral entry^17,18^. Although some single marker genes showed significant donor-related variation, a notable difference between nasal and lung-derived organoids was observed in markers for ciliated cells (*FOXJ1*,* SNTN*). This was expected as ciliated cells are more abundant in nasal epithelial tissue in comparison to lower airway epithelia^19^. Overall, the analysis demonstrated that airway organoids derived from different sources exhibit comparable cellular characteristics and mimic human airway tissue effectively. Although nasal organoids displayed smaller lumens and greater expression of ciliated cell markers, SNTN and FoxJ1, compared to lung-derived organoids, they showed stable comparable cellular composition.

**Fig. 3:**
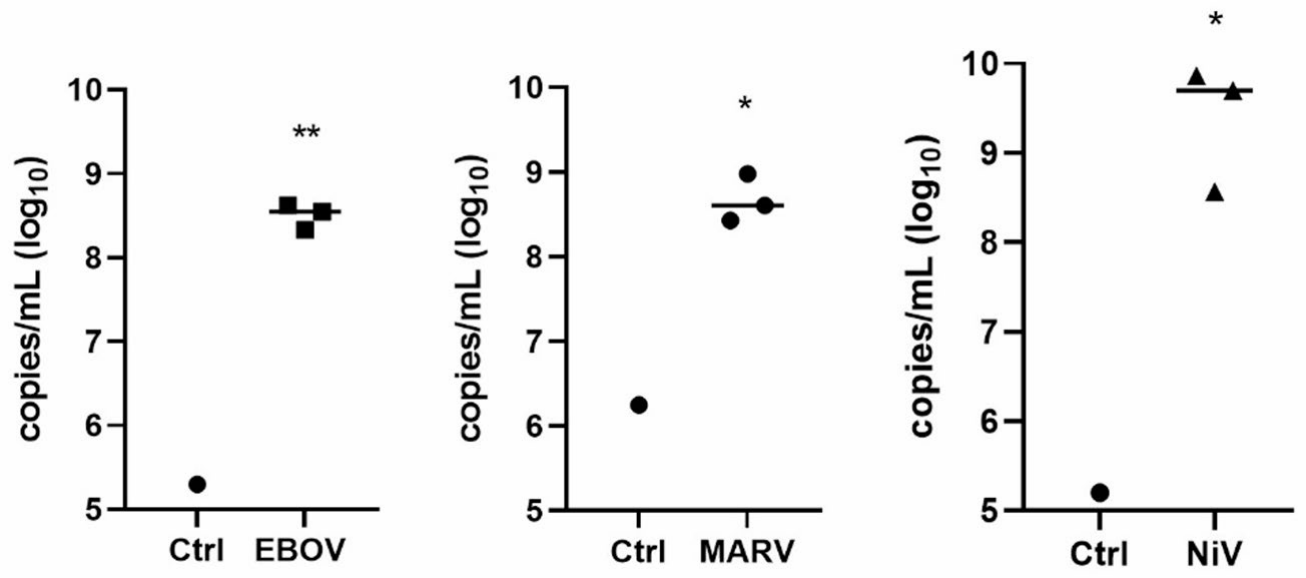
HNEpC-derived airway organoids (AOs) exhibit viral replication following infection with Ebola (EBOV), Marburg (MARV), and Nipah (NiV) virus. AOs were infected with EBOV, MARV and NiV. At 3 days post infection virus-specific copies per mL were quantified by qRT-PCR. All AOs were exposed to viruses for 1 h, controls were sampled after 5 min whereas remaining AOs were sampled after 3 d. Statistical significance was calculated using unpaired t-test (* = *p* ≤ 0.05; ** = *p* ≤ 0.01).

Next, we used nasal organoids for infection studies with BSL-4 viruses. This choice was driven by their commercial availability and therefore easy accessibility. Strikingly, the nasal-derived organoids showed high susceptibility to Ebola Zaire virus (EBOV), Marburg (MARV) and Nipah (NiV). These results mark the first proof-of-concept for using these respiratory organoids to study high risk pathogens in a controlled environment. Viral replication and release were monitored by quantifying the viral genome equivalents present within the organoids (Fig. 3). Following incubation with the initial virus input, a notable increase in viral genome equivalents was detected, indicating effective viral replication within the nasal-derived organoids.

## Discussion

In this study, we demonstrated that airway organoids from different origin exhibit remarkable consistency in cell type composition, yet maintain regional-specific tissue characteristics. While we used whole lung tissue as a baseline for gene expression in our airway organoids, it needs to be considered that it contains a diverse array of cell types, including those from the alveolar region, which limits its suitability as a control for studying gene expression in airway organoids. However, as we aimed to compare airway organoids derived from both the upper and lower airways, we chose whole lung tissue as a baseline control for both groups, rather than using separate region-specific controls.

The observed morphological differences between upper (nasal) and lower airway-derived organoids can be attributed to their differences in cellular composition and gene expression^20^. Despite the noted differences in gene expression, all organoids were highly susceptible to infection and replication of the BSL-4 viruses Ebola, Marburg and Nipah confirming their suitability as infection models. Due to the commercial availability of nasal cell-derived organoids, infection data was presented exclusively with this model. By providing insights into tissue-specific tropism and viral entry mechanisms, they may help uncover the molecular determinants that enable viruses like Ebola, Marburg or Nipah virus (EBOV, MARV, NiV) to selectively infect certain tissues. This could be particularly useful in future studies on the respiratory tract. Examining the expression levels of putative entry markers, like Niemann-Pick disease, type C1 (NPC1) for Ebola^21^ and Marburg virus and Ephrin B2 and B3 (EFNB2, EFNB3) for Nipah virus^22,23^, could pave the way for follow-up studies. Additionally, when supplemented with immune cells, airway organoids will allow real-time analysis of immune dynamics, making them well-suited models to study immune evasion strategies of these viruses^24^. Furthermore, these models may provide a promising platform to investigate vascular endothelial interactions, offering a future avenue to better understand how hemorrhagic viruses compromise blood–tissue barriers and drive vascular pathology^25,26^.

For therapeutic applications, airway organoids provide a robust platform to screen antiviral agents and evaluate tissue-specific drug efficacy and toxicity with potential for patient-tailored therapies in the context of precision medicine^17,27^. Finally, these models facilitate exploration of virus-host interactions and virus-induced metabolic shifts, which could identify novel therapeutic targets. The three-dimensional architecture of organoids provides an improved representation of host-virus interactions at cellular and molecular levels compared to conventional 2D cell culture systems^28–30^. Bridging the gap between 2D cell culture and animal models, organoids allow for physiologically relevant analysis of BSL-4 pathogens, enhancing our understanding of complex viral behaviour and therapeutic responses. Future research should focus on refining culture protocols and enhancing physiological relevance of organoids through the addition of vascularization, blood perfusion, and immune cell integration.

Since this study utilizes airway organoids, which primarily recapitulate the epithelium of the upper airway and therefore lack significant populations of lower airway epithelial cells, it has certain limitations in examining host lung cell tropism. Alternatively, alveolar organoids, which model the alveolar regions of the lower airways, could be used for infection experiments, enabling the study of virus interactions with alveolar type 1 and type 2 cells. While several human airway organoid infection models already exist^31^, this study contributes to the development of a valuable model system. It provides structural insights into comparability of different source materials and demonstrates its suitability for research in a high containment environment. In conclusion, our proof-of-concept study provides an easy to adapt method to study a range of BSL-4 viruses in an advanced organoid-based airway cell culture model using commercially available cells and published, robust culture protocols. This model is easily transferable to other respiratory viruses, making it a versatile platform for further viral research and therapeutic development.

## Materials and methods

### Patient samples

Patient-derived lung cells were provided by Lung Biobank Heidelberg, a member of the BioMaterialBank Heidelberg (BMBH) and the platform biobanking of the German Center for Lung Research (DZL). All patients signed an informed consent and study was approved by the ethics committees of Heidelberg and Berlin (S-270/2001 (biobank vote) and EA2/090/20 (study vote). All methods were performed in accordance with the relevant guidelines and regulations.

Briefly, cells were thawed into DMEM/Ham F-12 medium with ROCK inhibitor, centrifuged, and seeded in organoid medium (see Suppl. Table 2) into T25 flasks. Cells were later transferred into 6-well plates with Cultrex BME for 3D culturing, and medium was replaced weekly. For passaging, cells were enzymatically dissociated with TrypLE, centrifuged, and re-embedded in Cultrex BME (see Supplement for details on culture medium and methods).

### Organoid culture and maintenance

Organoid cultures were established from cryopreserved cells derived from resected lung lobe containing bronchial parts as well as lung parenchyma to receive different pulmonary stem cells (see Suppl. Table 1) following protocols from Zhou and Sachs^10,32^. Cells preserved in a 10% DMSO solution, were thawed in a 37 °C water bath and subsequently resuspended in (a 15 mL Falcon tube containing) 5 mL of pre-warmed DMEM/Ham F-12-Gibco medium (Gibco™ CAT#12634010), supplemented with 5µM Y-27632 (ROCK inhibitor; AbMole Bioscience CAT#M1817). Following centrifugation at 500 x RCF for 5 min at 4 °C, the pellet was resuspended in 10 mL of organoid medium (see Table 2) and seeded in a T25 flask. Cells were incubated overnight at 37 °C with 5% CO_2_. For subsequent steps of the 3D-culture, all components were kept on ice. The following day, cells from the T25 flask were collected, adherent cells were gently detached using a cell scraper, and transferred to a 15 mL Falcon tube coated with 0.1% BSA, kept on ice. Cells were then centrifuged at 500 x RCF for 5 min at 4 °C and the pellet was resuspended in an ice-cold mixture of 70% Cultrex Basement Membrane Extracts (BME, growth factor-reduced Type 2 basement membrane extract; R&D Systems CAT#3533-005-02) and 30% organoid medium. The cells were seeded as 70 µL BME droplet domes in 6-well plates, with each well containing three domes, and a total of one to three wells used, depending on the initial cell number. The plates were incubated for 30 min at 37 °C to allow solidification of the BME. Once solidified, 2 mL organoid medium was added to the domes and medium was changed twice a week. For subsequent passages and culture expansion, BME droplets were mechanically disrupted by washing over them with organoid medium using a P1,000 tip and then centrifuged at 500 x RCF for 5 min at 4 °C. This was followed by enzymatic digestion using TrypLE Express (Gibco™ CAT#12605010), supplemented with 10 µM ROCK inhibitor for 4–8 min at 37 °C. Cells were dispersed into a single-cell solution using a blunt needle (18G), followed by the addition of DMEM/Ham F-12 medium supplemented with ROCK inhibitor. After centrifugation as described earlier, cells were embedded again in 70% BME split in a 1:3 or 1:6 ratio, again in the form of droplets (each well containing three droplets). The organoids used for both characterization and infection experiments underwent two passages after thawing the cells. For each biological sample, a complete 6-well plate was utilized, comprising a total of 18 droplets.

### Viruses and infection

Ebola Zaire-GFP^33^, Ebola Zaire (Makona), and Marburg virus (Musoke) were kindly provided by Prof. Dr. Stephan Becker, Institute for Virology, Philipps-University Marburg, Germany, and Nipah virus (Malaysia) was kindly provided by Heinz Feldmann, Rocky Mountain Laboratory, Hamilton, Montana, USA. All viruses were propagated in green monkey kidney epithelial Vero E6 cells (American Type Culture Collection, Manassas, VA, CAT#CCL-1586). The viral titer was determined using the Spearman and Kärber method, specifically the TCID_50_/mL (Tissue Culture Infectious Dose 50 per milliliter) assay^34^.

The experiments involving risk group 4 viruses were conducted at the BSL-4 facility of the Robert Koch-Institute in Berlin, Germany, strictly following standard operating protocols for handling infectious agents. Organoids derived from commercially available airway epithelial cells sourced from nasal swabs were cultured for approximately 20 d prior to infection as previously described. Organoids grown in 18 × 70 µL domes were collected into 15 mL tubes and centrifuged at 500 × RCF for 5 min, leaving a liquid pellet containing the organoids. The supernatant was discarded and BME was removed by incubating the pellet in 3 mL cell recovery solution for 45 min at 4 °C with resuspension every 15 min, followed by another centrifugation step. Subsequently, 1260 µL of 70% BME, diluted in organoid medium, was mixed with 25 µl EBOV (TCID_50_/mL = 2.94 × 10^7^); 18.4 µl MARV (TCID_50_/mL = 3.98 × 10^7^) and 11.7 µl NiV (TCID_50_/mL = 6.3 × 10^7^), respectively, to reach equal levels of infection doses; organoids were resuspended in this mix avoiding bubble formation on ice. This mixture was then seeded as droplets in 6-well LabTeks plates and incubated at 37 °C for 1 h. Once solidified, the BME droplets were maintained for the specified durations in a previously reported medium^10^. Organoids were collected and pelleted as described above in this section and organoids were re-seeded in 70% BME in the absence of viral input. Controls were treated simultaneously, but organoids were sampled after 5 min to ensure only input virus will be quantified. *RNA Purification*,* cDNA Synthesis*,* and qPCR.* This study employed two distinct quantitative polymerase chain reaction (qPCR) protocols. The initial protocol, which utilized a two-step qPCR method, was applied for organoid characterization, while a one-step qPCR approach was implemented for the infection experiments. For the two-step qPCR, 18 organoid domes embedded in BME were collected, centrifuged at 500 x RCF for 5 min at 4 °C. Subsequently, the pellet was resuspended in 700 µl of Trizol Reagent (Zymo Research CAT#R2050-1-200) and stored at -80 °C until further processing. RNA extraction was performed using the Zymo Quick-RNA Microprep Kit (Zymo Research CAT#R1050) following the manufacturer`s instructions. RNA concentration was assessed using Tecan’s NanoQuant Plate. Synthesis of cDNA was employed using the High Capacity RNA-to-cDNA kit (Applied Biosystems-Thermo Fisher Scientific CAT#4387406). 20 µL of the reaction mixture were pipetted into ice-cold RNase-free PCR tubes. During the incubation step, the mixture was heated to 70 °C for 5 min without the addition of RT Enzyme Mix, facilitating denaturation and allowing for initial annealing. The RT program encompassed 60 min at 37 °C, 5 min at 95 °C, and a final step at 4 °C. After cDNA synthesis, samples were diluted with nuclease-free water to a concentration of 2 ng/µL in preparation for qPCR analysis. Real-time quantification was carried out using 10 ng (5 µL) of cDNA with SYBR Green LUNA Universal qPCR (New England BioLabs CAT#M3003) on a Bio-Rad CFX96 qPCR real-time thermocycler. For the one-step qPCR, 18 embedded organoids were collected, centrifuged at 500 x RCF for 5 min at 4 °C. BME was removed by incubating the pellet in 3 mL cell recovery solution for 45 min at 4 °C with resuspension every 15 min. Following another centrifugation step the supernatant was removed and organoids were lysed in 300 µL RLT buffer and RNA was isolated following the manufacturer`s instruction (Qiagen CAT# 74104). RNA samples for one-step qPCR analysis were diluted with nuclease-free water to 2 ng/µL. Real-time quantification was performed using the SYBR Green One-Step RT-qPCR Kit (New England BioLabs CAT#E3005) with 10 ng of RNA on a Bio-Rad CFX96 qPCR Real-Time thermocycler. Primer sequences are listed in the Suppl. Table 3.

### Immunofluorescence staining and microscopy

Organoids were collected and centrifuged at 500 x RCF for 5 min at 4 °C. Thereafter, in order to prevent organoid damage, centrifugation was avoided and, cells were allowed to settle naturally. The pellet containing organoids and BME were incubated in cell recovery solution (Corning CAT#CLS354253) for 45 min at 4 °C to dissolve the extracellular matrix. During the incubation organoids were diligently resuspended every 10 min using a 0.1% BSA-coated pipette tip. After settling, supernatant was aspirated and the pellet was washed with PBS to eliminate any residual supernatant. Subsequently, the organoids were fixed in 4% paraformaldehyde (PFA, ROTH CAT#0335.1) for 45 min at 4 °C. During the fixation process, organoids were gently resuspended every 10 min with a BSA-coated pipette tip. Following fixation, two additional PBS washes were performed. The organoids were then incubated in permeabilization buffer (1xTBS, 0.25% TritonX-100, 0.1 M glycine for 20 min) at room temperature. Following settling, the permeabilization buffer was carefully removed and samples were incubated in blocking buffer (1xTBST, 0.02% Triton-X100, 3% BSA, 1% normal goat serum) for 1 h at room temperature. Primary antibodies (see Table 4) were diluted in 900 µl of blocking buffer as indicated. The organoids were incubated overnight with the primary antibodies at 4 °C, washed three times with washing buffer (1xTBS-T containing 0.05% Tween and 0.02% Triton-X100) and allowed to settle before removing the supernatant. Afterwards, the organoids were incubated with the secondary antibodies (see Table 4) in blocking buffer, DAPI for nuclear staining, and phalloidin for actin filament staining at room temperature for approximately 2 h. Following staining, three washes with washing buffer, as described above, were carried out, followed by a final wash with Milli-Q water. Finally, the organoids were gently resuspended in approximately six to eight drops of non-hardening mounting medium (Ibidi, CAT#50001) and placed onto microscope slides. During the mounting process, spaces were left between the slide and the coverslip to prevent organoid compression. These spaces were sealed with nail polish to ensure proper preservation. Image acquisition was performed using an inverted fluorescence confocal microscope (Leica STELLARIS 8) with a 20x objective. The imaging software employed was LAS X^35^ with image resolution set to 1024 × 1024 pixels, and subsequent image editing was conducted using ImageJ software^36^. Full antibody list can be found in Table 4 (Suppl.)

### Data analysis

The qRT-PCR data were subjected to analysis using the ΔΔCt method^37^. For assessing the expression changes of the gene of interest (GOI), the mean Cq values of the biological replicates for the GOI were initially computed. These Cq values were then normalised against the reference gene ß-Actin. The obtained results from the control (lung tissue) and donor samples were juxtaposed, and ΔΔCq values were computed.

Statistical analysis was performed using GraphPad Prism^38^. Ordinary one-way ANOVA was employed for qPCR analyses pertaining to organoid characterization, comparing means across more than two populations, assuming normal distribution and equal variance for all tests. Boxplots show the median and interquartile range, whiskers represent the highest and lowest values, outliers are plotted as dots. For analysis of qPCR data showing viral replication, unpaired t-test was performed. The presented p-values are as follows: ns (not significant) = *p* > 0.05; * = *p* ≤ 0.05; ** = *p* ≤ 0.01; *** = *p* ≤ 0.001; **** = *p* ≤ 0.0001.

## Supplementary Information

Below is the link to the electronic supplementary material.

Supplementary Material 1

Supplementary Material 2

Supplementary Material 3

## Data Availability

All data generated or analysed during this study are included in this published article (and its Supplementary Information files).
