# Supplementary material for: Human airway organoids as a versatile model to study BSL-4 virus replication and pathogenesis
Source: Sci Rep. 2026 Mar 27;16:10517. doi: 10.1038/s41598-026-45813-6 (PMC13035905; doi:10.1038/s41598-026-45813-6)
Supplement: Supplementary file 2 — Supplementary Material 2 [file 41598_2026_45813_MOESM2_ESM.docx]

Human Airway Organoids as a Versatile Model to study BSL-4 Virus replication and pathogenesis

**Joo-Hee Wälzlein, Sebastian Reusch, Jenny Ospina-Garcia, Ruth Olmer, Marc A. Schneider,
Laura V. Klotz, Christian Klotz and Susann Kummer**

**SUPPLEMENTARY INFORMATION**

**Material and Methods**

***Organoid cultures and maintenance***

**Table 1: Sources of material used for airway organoid generation**

| **Material** | **Source** | **Description** |
| --- | --- | --- |
| Donor 1 | Lung Biobank Heidelberg, member of the accredited Tissue Bank of the National Center for Tumor Diseases (NCT) Heidelberg, the Biomaterial Bank Heidelberg, and the Biobank platform of the German Center for Lung Research (DZL) | Lung biopsy of non-cancerous tissue from a 70-year-old, non-smoking patient diagnosed with pulmonary carcinoid |
| Donor 2 | Leibniz Research Laboratories for Biotechnology and Artificial Organs (LEBAO), Biomedical Research in Endstage and Obstructive Lung Disease Hannover (BREATH), German Center for Lung Research (DZL), Department of Cardiothoracic, Transplantation and Vascular Surgery Hannover Medical School, Hannover, Germany) | Lung biopsy of non-cancerous tissue from a 50-year-old patient diagnosed with fibrosis |
| HNEpC | PromoCell (CAT #C-12620, Lot 475Z023) | Primary human nasal epithelial cells isolated from normal human nasal mucosa |
| Lung tissue | DZL Heidelberg |  |

**Table 2: Complete organoid medium**

| **Medium supplement** | **Final concentration** |
| --- | --- |
| Advanced DMEM/F12 | 1x |
| R-Spondin 1 | 500 ng/mL |
| FGF-7 | 25ng/mL |
| FGF-10 | 100ng/mL |
| Noggin | 100ng/mL |
| A83-01 | 500nmol/L |
| Y-27632 | 5µmol/L |
| SB202190 | 500nmol/L |
| N-Acetylcysteine | 1.25mmol/L |
| Nicotinamide | 5mmol/L |
| B27 | 1x |
| Pen/Strep/Glutamine | 100U/mL; 100µg/mL; 1x |
| Hepes | 10mmol/L |
| Primocin | 50µg/mL |

***RNA purification, reverse transcription and qPCR***

The primer pairs (see table 3) for the different markers were validated and efficiency of primers was calculated as described [26].

**Table 3:** **Primer used for qRT-PCR**

| **Target** | **Sequence** | **Gene ID** |
| --- | --- | --- |
| **KRT5** | Fw: CCAAGGTTGATGCACTGATGG  Rev: TGTCAGAGACATGCGTCTGC | 3852 |
| **MUC5AC** | Fw: CAGCACAACCCCTGTTTCAAA  Rev: GCGCACAGAGGATGACAGT | 4586 |
| **SCGB1A1** | Fw: TCCTCCACCATGAAACTCGC  Rev: AGGAGGGTTTCGATGACACG | 7356 |
| **FOXJ1*** | Fw: AGATCCCACCTGGCAGAATTCAA  Rev: CCGAGGCACTTTGATGAAGC | 2302 |
| **SNTN** | Fw: TGTATGCACAGTACCCAGGAC  Rev: AGCAGTGGTGGCAATAGCTTT | 132203 |
| **MUC5B** | Fw: GCCTACGAGGACTTCAACGTC  Rev: CCTTGATGACAACACGGGTGA | 727897 |
| **ITGA6*** | Fw: ATGCACGCGGATCGAGTTT  Rev: TTCCTGCTTCGTATTAACATGCT | 3655 |
| **TMPRSS2*** | Fw: CAAGTGCTCCAACTCTGGGAT  Rev: AACACACCGATTCTCGTCCTC | 7113 |
| **ACE2*** | Fw: CAAGAGCAAACGGTTGAACAC  Rev: CCAGAGCCTCTCATTGTAGTCT | 59272 |
| **GAPDH*** | Fw: CTCCTGTTCGACAGTCAGCC  Rev: CCCAATACGACCAAATCCGTTG | 2597 |
| **ß-Actin*** | Fw: CATGTACGTTGCTATCCAGGC  Rev: CTCCTTAATGTCACGCACGAT | 60 |
| **RPS18*** | Fw: AGTTCCAGCACATTTTGCGAG  Rev: TCATCCTCCGTGAGTTCTCCA | 6222 |
| **GFP** | Fw: GAGCGCACCATCTTCTTCAA  Rev: CTGCTTGTCGGCCATGATATAG | 25339618 |
| **Ebola Zaire, VP30** | Fw: ACT CCT ACT AAT CGC CCG TAA G  Rev: ATC AGC CGT TGG ATT TGC T  Probe: CACCCAA+GGACTCGC |  |
| **Marburg, NP** | Fw: GTCCTCAGCCAGAAACGAGA  Rev: ACCGTTACTTCCACAGGTGT  Probe: TCACAGAATCGGGTGTCACAGTCGT |  |
| **Nipah Malaysia, NP** | Fw: gttcaggctagagaggcaaaattt  Rev: ccccttcatcgatatcttgatca  Probe: ctgcaggaggtgtgctcattggagg |  |

* Sequence taken from PrimerBank, Harvard Medical School.

***Immunofluorescence staining and laser scanning microscopy.***

**Table 4: Primary and secondary antibodies employed for immunofluorescence staining**

| **Antibody** | **Company** | **Order Number** | **Dilution** | **Host** |
| --- | --- | --- | --- | --- |
| ***Primary Antibodies*** | | | | |
| Anti-CC10-E11 | Santa Cruz | sc365992 | 10ug/ul | Mouse |
| Anti-Mucin 5AC | Thermo Fisher | MA5-12178 | 1:100 | Mouse |
| Anti-acetylated tubulin | Sigma Aldrich | T6793 | 1:100 | Mouse |
| Anti-Cytokeratin 5 | Biolegend | 905501 | 1:100 | Rabbit |
| ***Secondary Antibodies*** | | | | |
| αMouse IgG (H+L) | Thermo Fisher (Alexa Fluor647) | A28181 | 1:250 | Goat |
| αRabbit IgG (H+L) | Thermo Fisher (Alexa Fluor647) | A27040 | 1:500 | Goat |
| Phalloidin iFluor | Abcam (iFluor 488) | ab176753 | 1:1,000 | - |
| DAPI | Abcam | ab228549 | 1:500 | - |
